# THE RELATIONSHIP BETWEEN ORAL HEALTH AND COGNITIVE FUNCTION: EVIDENCE FROM THE LASI

**DOI:** 10.1093/geroni/igad104.2957

**Published:** 2023-12-21

**Authors:** Xi Pan, Uma Kelekar, T Muhammad

**Affiliations:** Texas State University, San Marcos, Texas, United States; Marymount University, Arlington, Virginia, United States; International Institute for Population Sciences, Mumbai, Maharashtra, India

## Abstract

The present study aimed to examine the association between oral health and cognitive function among older adults in India. Data for the study were selected from the Longitudinal Aging Survey of India (LASI, 2017-2019) and the LASI Diagnostic Assessment of Dementia (LASI-DAD) and 4,096 respondents aged 60 and above were included. Oral health was measured by self-reported physician-diagnosed oral health problems including presence of painful teeth, ulcers for more than two weeks, bleeding gums, swelling gums, loose teeth, dental cavities or caries, soreness or cracks in the corners of the mouth, or other problems as well as edentulism (i.e., losing all natural teeth, some natural teeth, or no teeth). A higher number of these problems indicated a more complex oral health condition. Cognitive function was measured by the standardized total score of all cognitive tests in the Harmonized Cognitive Assessment Protocol (HCAP). Binomial logistic regression models were used to analyze the relationship between the diagnosis of oral health problems and cognitive function. Results showed that having one or more oral health problems was associated with lower scores on cognitive function (β=-0.16, p=0.04), after controlling for sociodemographic characteristics and health status. There was no significant association between edentulism and cognitive function. Findings of the study suggest that oral health is integral to long-term overall health, specifically cognitive function. Our findings highlight the importance of educating older individuals about the connection between oral health and cognitive function along with the need for broadening their access to oral health care in India.

